# The highly sensitive brain: an fMRI study of sensory processing sensitivity and response to others' emotions

**DOI:** 10.1002/brb3.242

**Published:** 2014-06-23

**Authors:** Bianca P Acevedo, Elaine N Aron, Arthur Aron, Matthew-Donald Sangster, Nancy Collins, Lucy L Brown

**Affiliations:** 1 Department of Psychological and Brain Sciences, University of CaliforniaSanta Barbara, California; 2 Department of Psychology, Stony Brook UniversityNew York, New York; 3 Monmouth UniversityMonmouth County, New Jersey; 4 Department of Neurology, Albert Einstein College of MedicineBronx, New York

**Keywords:** Emotion, empathy, highly sensitive person, magnetic resonance imaging, mirror neurons, sensory processing sensitivity

## Abstract

**Background:**

Theory and research suggest that sensory processing sensitivity (SPS), found in roughly 20% of humans and over 100 other species, is a trait associated with greater sensitivity and responsiveness to the environment and to social stimuli. Self-report studies have shown that high-SPS individuals are strongly affected by others' moods, but no previous study has examined neural systems engaged in response to others' emotions.

**Methods:**

This study examined the neural correlates of SPS (measured by the standard short-form Highly Sensitive Person [HSP] scale) among 18 participants (10 females) while viewing photos of their romantic partners and of strangers displaying positive, negative, or neutral facial expressions. One year apart, 13 of the 18 participants were scanned twice.

**Results:**

Across all conditions, HSP scores were associated with increased brain activation of regions involved in attention and action planning (in the cingulate and premotor area [PMA]). For happy and sad photo conditions, SPS was associated with activation of brain regions involved in awareness, integration of sensory information, empathy, and action planning (e.g., cingulate, insula, inferior frontal gyrus [IFG], middle temporal gyrus [MTG], and PMA).

**Conclusions:**

As predicted, for partner images and for happy facial photos, HSP scores were associated with stronger activation of brain regions involved in awareness, empathy, and self-other processing. These results provide evidence that awareness and responsiveness are fundamental features of SPS, and show how the brain may mediate these traits.

## Introduction

Sensory processing sensitivity (SPS) is proposed to be an innate trait associated with greater sensitivity (or responsivity) to environmental and social stimuli (e.g., Aron et al. 2012). Originally measured in human adults by the Highly Sensitive Person (HSP) scale (Aron and Aron 1997), SPS is becoming increasingly associated with identifiable genes, behavior, physiological reactions, and patterns of brain activation (Aron et al. 2012). A functionally similar trait—termed responsivity, plasticity, or flexibility (Wolf et al. 2008)—has been observed in over 100 nonhuman species including pumpkinseed sunfish (Wilson et al. 1993), birds (Verbeek et al. 1994), rodents (Koolhaas et al. 1999), and rhesus macaques (Suomi 2006).

Sensory processing sensitivity is thought to be one of two strategies that evolved for promoting survival of the species (Aron and Aron 1997; Wolf et al. 2008). By being more responsive to their environments, these more sensitive organisms have an enhanced awareness of opportunities (e.g., food, mates, and alliances) and threats (e.g., predators, loss of status, competitors), and thus may be more ready to respond to emerging situations. This survival strategy is effective as long as the benefits of increased sensitivity outweigh the costs (such as increased cognitive and metabolic demand). In addition to potential costs, those with the sensitive survival strategy will always be in a minority as it would cease to yield special payoffs if it were found in a majority (Wolf et al. 2008).

Humans characterized as high SPS (or HSP) are likely to “pause to check” in novel situations (Aron and Aron 1997; Aron et al. 2012), show heightened awareness of and attention to subtle stimuli, and appear to be more reactive to both positive and negative stimuli (Jagiellowicz 2012). This combination supports a tendency to process stimuli more elaborately and learn from the information gained, which may be useful in the present moment and when applied to future situations. In contrast, those low in SPS pay less attention to subtle stimuli, approach novel situations more quickly, are less emotionally reactive, and behave with less reference to past experiences.

At least two brain imaging studies have examined the attentional and perceptual aspect of SPS in humans, using the HSP scale as a measure of SPS. One study asked individuals to notice subtle differences in photographs of landscapes and found that those with greater SPS showed stronger activation in brain regions for visual and attention processing compared to those low in SPS (Jagiellowicz et al. 2011). A second study, by Aron et al. (2010), compared individuals from East Asia and the United States and showed that SPS moderates the effect of culture on neural responses to culturally relevant cognitive tasks. There was a strong cultural difference in the activation of brain regions associated with attention such that low-SPS participants showed greater activation when completing tasks that were inconsistent with their cultural context. However, among those high in SPS, there was no cultural difference in brain activation in regions associated with attention. These findings suggest that high- (vs. low-) SPS individuals focus on the task itself independent of other factors.

Studies have also identified genetic polymorphisms' association with SPS. One of these studies (Licht et al. 2011) found an association with polymorphisms of the low-expressing, short (S) variant of the repeat length polymorphism 5-HTTLPR (serotonin transporter, 5-HTT, linked polymorphic region). There is some evidence that carriers of the S-allele (either two shorts or the short and long combination) are more likely to be depressed in response to stressful life events (Homberg and Lesch 2011). Not surprisingly, since “genetically driven deficient serotonin transporter (5-HTT) function would not have been maintained throughout evolution if it only exerted negative effects” (Homberg and Lesch 2011, p. 513), increasing research suggests that the S-allele also has advantages (for a review see Homberg and Lesch 2011). For example, it has been associated with superior performance on perceptual tasks—more risk aversion when there was a low probability of winning, but greater risk seeking when there was a high probability of winning; longer reflection before making difficult choices and better performance on a delayed pattern recognition task (Roiser et al. 2006; Jedema et al. 2009). The role of the S-allele in a social context has also been studied (e.g., Way and Gurbaxani 2008; Way and Taylor 2010). For example, marital partners with the S-allele were more affected after a marital discussion by their partner's positive or anxious prediscussion mood (Schoebi et al. 2012). In another study of the possible genetics behind SPS, researchers (Chen et al. 2011) sought to find something closer to the strong associations between genes and traits that are predicted by twin studies but not being found with single gene research. They considered essentially all the genes (98) with polymorphisms that affect the dopamine system, and chose a trait, SPS, “deeply rooted in the nervous system” (p. 1). Employing a multistep approach (ANOVA followed by multiple regression and permutation), they found that 15% of the variance of HSP scale scores were predicted by a set of 10 loci on seven genes.

Evolutionary theories of SPS are still developing and vary (e.g., Wolf et al. 2008, 2011; Ellis et al. 2011; Aron et al. 2012; Pluess and Belsky 2013), but all emphasize that there are advantages to it, many of them being social. For example, responsiveness to others' needs is essential for stabilizing cooperative relationships and trust in humans and other species (e.g., McNamara et al. 2009). Indeed, SPS—whether it is measured by questionnaires, physiological measures, behavioral observations, or genetic markers—confers benefits to individuals in “good-enough” social environments but vulnerability to negative outcomes in poor ones (e.g., Belsky and Pluess 2009; Pluess and Belsky 2013).

At least two experimental studies relevant to SPS support the idea that it is associated with responsiveness to both positive and negative stimuli. In one experiment, participants were led to believe that they did well or poorly on a general aptitude test (Aron et al. 2005a, Study 4). Those high (vs. low) on SPS had more negative affect when they thought they had low scores on the test, but when they thought they had high scores there was a nonsignificant crossover. In another study, Jagiellowicz (2012) examined the association between SPS (as measured by the HSP scale) and emotional responses to positive and negative images from the International Affective Picture System. High- (vs. low-) SPS individuals rated emotional pictures (especially positive ones) as significantly more positive or negative and tended to respond faster to positives. Also, high- versus low-SPS individuals reporting positive parenting in early childhood reported more arousal to positive pictures. However, the mechanisms by which positive (or negative) social experiences may potentiate the effect of SPS on emotional reactivity have not yet been studied. Moreover, given that SPS is responsive to both positive and negative social environments, we examined whether highly sensitive individuals might show stronger neural responses in predicted brain regions to both positive and negative social stimuli.

### The present study

As briefly reviewed above, SPS theory and research suggest that greater awareness and responsiveness to others' moods and emotions are central features of being highly sensitive. However, no study has measured the link between SPS and neural reactivity in response to others' emotional states. Thus, the primary goal of this study was to investigate individuals' brain activity in response to close others' and strangers' positive and negative facial expressions as a function of SPS. To accomplish this goal, we adapted a paradigm used in previous research in which mothers' brain activity was measured while viewing happy and sad facial images of their infants and of others' infants (Strathearn et al. 2008). Using fMRI we examined the neural activations of individuals in intimate relationships, who were recruited as part of a larger longitudinal study on marriage (Acevedo 2014). Participants were scanned twice approximately 1-year apart to provide a replication of results.

At Time 1 (T1), we varied two factors in a within-subjects design (a) the target (partner vs. stranger) and (b) emotional expressions (happy vs. sad). By varying partner versus stranger photos, we were able to explore whether brain activations of individuals higher on SPS, as measured by the HSP scale (Aron and Aron 1997), would be stronger in regions relevant to responding to emotions of close others versus strangers; particularly in brain regions reflecting awareness, empathy, and readiness to act. At Time 2 (T2), we replicated T1 and included an emotionally neutral facial expression condition. This additional condition enabled us to examine more directly the extent to which SPS would be differentially associated with neural reactivity in response to positive or negative facial expressions versus neutral ones.

## Method

### Participants

Participants were recruited by flyers, newspaper, and Internet advertisements as part of a larger study of newlyweds and engaged couples in the Santa Barbara, CA, community (Acevedo 2014). All participants provided informed consent and received payment for their participation. The study was approved by the human subjects committees at the University of California, Santa Barbara (UCSB) and Albert Einstein College of Medicine.

Individuals were screened for eligibility criteria (e.g., relationship status, age 22–40 years, nonuse of antidepressants, and fMRI contraindications), medications, surgeries, and overall health. Approximately 34% of individuals screened were excluded for not meeting criteria. No participant included in the study reported a history of any disorders (e.g., anxiety, personality disorders, social disorders) or use of medications that might bias responses to the HSP scale. In addition, as in other studies, neuroticism was partialed out of the HSP scale scores because neuroticism is correlated with HSP scale scores (e.g., Aron et al. 2005a) and answers to negative questions on the scale can be shifted in a more negative direction by high neuroticism. Thus, results reported herein are not confounded with neuroticism.

Participants completed data collection (fMRI and surveys) at two visits, about 1-year apart. At T1, scanned participants were 18 (10 women) healthy, right-handed individuals; age 21–32 years (*M* = 27.50, SD = 3.13), in established relationships (*M* = 4.30 years, SD = 3.18), and had completed roughly 16 years (SD = 1.09) of education. The ethnic/racial composition of the sample was 72% Caucasian, 17% Asian, and 11% Hispanic. At T2, 13 (7 women) of the original 18 participants completed fMRI scanning, with age ranging from 22 to 33 years (*M* = 28.38, SD = 3.40); and average relationship lengths of 5.88 years (SD = 2.88).

### Questionnaires

Participants completed a battery of questionnaires, including an 11-item version of the HSP scale (Aron and Aron 1997), of which the full 27-item measure has been found to be a unidimensional with alphas of 0.65–0.85 across numerous samples (e.g., Meyer et al. 2005; Benham 2006; Hofmann and Bitran 2007). Sample items include “Are you easily overwhelmed by things like bright lights, strong smells, coarse fabrics or sirens close by?” “Do other people's moods affect you?” “Do you become unpleasantly aroused when a lot is going on around you?” Scores in this study ranged from 1 to 7 (T1: *M* = 3.97, SD = 1.32; T2: *M* = 4.08, SD = 1.18). The mean and distribution of SPS scores in the present sample were nearly identical to those found in larger studies of HSPs within normative populations (e.g., Aron and Aron 1997) and with the 11-item version of the HSP scale (e.g., Aron et al. 2010). In addition, the correlation between T1 and T2 HSP scores in the present sample was strong (*r* = 0.99), indicating high test–retest reliability.

Participants also completed a two-item measure of neuroticism (negative affectivity) used in previous studies of SPS (e.g., Aron et al. 2005a; Jagiellowicz et al. 2011) describing themselves on a scale from 1 (*strongly disagree*) to 7 (*strongly agree*) on the items: (1) anxious, easily upset and (2) calm, emotionally stable (reverse scored). In the present sample, *r*_T1T2_ = 0.82; T1: *M* = 2.72, SD = 1.09; T2: *M* = 2.38, SD = 1.09; *r* with SPS measure; T1 = 0.28, T2 = 0.25; both *ns*. This measure was included, as in the previous studies, to provide a control for negative affectivity, which, if not controlled for, distorts HSP scale scores.

### Stimuli

#### Partner and stranger facial photos

We presented digitized color photographs of participants' romantic partners and of strangers (control) displaying positive and negative facial expressions using Presentation software (Psychological Software Tools, Inc., Pittsburgh, PA). Strangers' images were matched to each participant's partner by sex, approximate age, ethnicity, and attractiveness.

#### Context-based descriptions

Because context plays a central role in emotion processing and regulation (e.g., Gross and John 2003), each facial image was preceded by a corresponding contextual description such as “This person is feeling very happy because something wonderful has happened to them” or “This person is feeling very sad and they are suffering because something terrible has happened to them.” This was done to enhance emotion-specific effects and reduce cognitive ambiguity as suggested by emotion research experts (e.g., McRae et al. 2011).

#### Countback task

After each photo (of all four types), participants were shown a four-digit number and instructed to mentally count back by 7s. Following Aron et al. (2005b), the countback task served as an attentional control and to reduce carry-over effects between stimuli. It is possible that this task creates stress or negative emotion (Wang et al. 2005), but such effects should balance out when comparisons are made across conditions since the same task was used after all four photo types.

#### Emotion ratings

While still in the scanner, but after completing the scanning session, participants provided emotion ratings for each photo they viewed during the experiment. The instructions read “Now you will see a series of emotion words. Please rate how you felt while viewing images of X” (where X is either [a] PARTNER SMILING, [b] PARTNER FROWNING, [c] STRANGER SMILING, or [d] STRANGER FROWNING appeared). A series of positive (e.g., joy) and negative (e.g., sadness) emotion words appeared on the screen and participants were asked to make responses via a button response box on a scale from 1 (*not at all*) to 4 (*a great deal*).

#### Attractiveness ratings of photos by independent raters

All photos were rated for facial attractiveness by independent coders (matched to participants by age, sex, and demographics) to verify that partner and stranger images did not differ systematically in terms of attractiveness.

### Design and procedure

Approximately 1 week prior to scanning participants were provided with a packet of questionnaires, which they completed and brought with them to the scanning session. Scanning was performed at the Brain Imaging Center (BIC) at the University of California, Santa Barbara. Just prior to scanning, participants were given a verbal description of the study and instructed to read the contextual descriptions, view each photo, and allow themselves to think and feel any response it might elicit. Once participants indicated that they were ready, they were oriented to the scanner. Correct positioning was confirmed via localized anatomical scans. At T1, the fMRI scanning block consisted of four conditions: partner happy, partner sad, stranger happy, and stranger sad. At T2 fMRI scanning, we included two additional conditions: partner neutral and stranger neutral. The conditions were randomized. Each condition included the following stimuli in sequential order: contextual description (6-s), face image (12-s), and a countback task (12-s). Each trial was presented randomly six times. Immediately after scanning, participants provided emotion ratings.

### Data acquisition and analysis

MRI scanning was performed using a 3T Siemens (Brain Imaging Center at the University of California, Santa Barbara, CA) magnetic resonance imaging system with a NOVA head coil. First, anatomical scans were obtained followed by a circle localizer. Next, functional images were obtained and the first four volumes were discarded to allow for proper calibration. A repetition time TR of 2000-msec was used with a TE of 30-msec, a 90° flip angle, and a voxel size for functional images of 3 × 3 × 3 mm collected in volumes of 30; 3-mm axial slices (0-mm gap) covering the whole brain.

Data were analyzed using SPM5 (http://www.fil.ion.ucl.ac.uk/spm). For preprocessing, functional EPI volumes were realigned to the fist volume, smoothed with a Gaussian kernel of 6 mm, and then normalized to the T1.nii image template. During normalization, we resliced voxels to 3 × 3 × 3 mm. No participant showed movement greater than 3 mm (whole voxel). After preprocessing, contrasts were created (e.g., partner happy vs. stranger happy) followed by regression analyses examining the associations between each contrast with HSP (controlling for neuroticism). Analyses were carried out using a mixed effects general linear model, with participants as the random-effects factor and conditions as the fixed effect. Following standard procedures using the HSP scale (as noted earlier), HSP scale scores were computed controlling for neuroticism in all conditions used for brain activation correlations.

#### Region-of-interest analyses

For all conditions, we utilized regions of interest (ROIs) based on previous fMRI studies of SPS (e.g., Aron et al. 2010; Jagiellowicz et al. 2011), empathy (Lamm et al. 2011), emotional memory encoding (Murty et al. 2011), responses to romantic partners (Singer et al. 2004), and emotional faces (e.g., Aharon et al. 2001; Fan et al. 2011; Fusar-Poli et al. 2009). A list of ROIs with Talairach coordinates as referenced in seed papers are provided in Table S1. We converted the Talairach to MNI coordinates to be consistent with the SPM T1.nii template. We adopted a false discovery rate (FDR) for multiple comparisons correction (Genovese et al. 2002) with a threshold of *P ≤* 0.05 and report the *P*(uncorrected) values as we conducted 63 small volume corrections thus increasing the likelihood of positive results. The ROIs occupied a 10-mm radius with a 3-voxel minimum. We used a 3 voxel minimum rather than a larger number to detect small regions in the brainstem, for example, but also cortical regions of functionally significant activation are not necessarily as large as 10 or 15 voxels. Anatomic regions were confirmed with the *Atlas of the Human Brain* (Mai et al. 2008).

#### Exploratory, whole-brain analysis

For each contrast, we also conducted exploratory, whole-brain analyses applying a threshold of *P ≤* 0.001 (uncorrected for multiple comparisons) with a spatial extent of ≥15 contiguous voxels.

## Results

### Behavioral results

#### Emotion ratings

**Figure 1:**
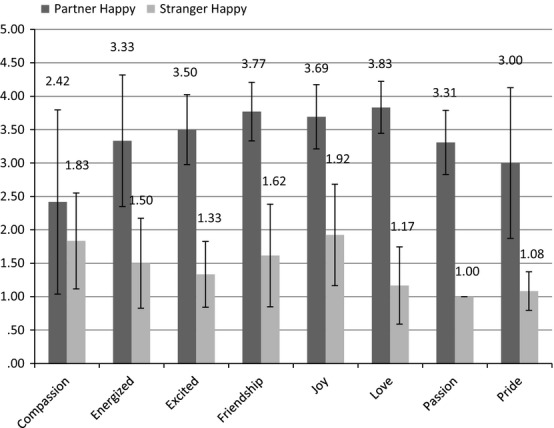
Postscan emotion ratings. The *y*-axis indicates the mean and standard error for the emotion intensity ratings given by participants while they were in the scanner at Time 2 for the partner happy versus stranger happy condition. Scores based on 1–4 scale, 1 = *not at all* and 4 = *a great deal*.

**Figure 2:**
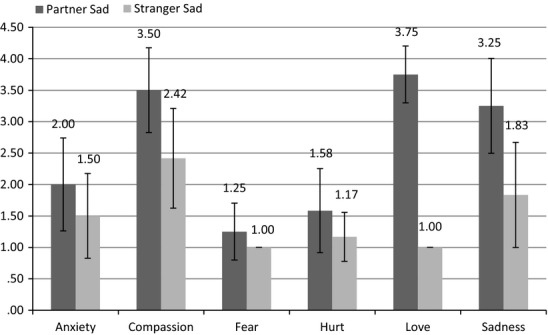
Postscan emotion ratings. The *y*-axis indicates the mean and standard error for the emotion intensity ratings given by participants while they were in the scanner at Time 2 for the partner sad versus stranger sad condition. Scores based on 1–4 scale, 1 = *not at all* and 4 = *a great deal*.

We conducted a series of paired *t*-tests to confirm that our manipulation elicited the intended affective responses. Results from paired *t*-tests showed that positive emotion (e.g., joy) ratings were significantly greater for partner happy images versus stranger happy images at T1 and T2 (both *P*s < 0.01). Paired *t*-tests also showed that participants reported significantly greater intensity of anxiety, compassion, fear, love, hurt, and sadness in response to partner sad images versus stranger sad images at T1 and T2 (all *P*s < 0.01). (See Figs. 1, 2 for T2 results. Note that although there was substantial between-subject variance for many emotion ratings, the within-subject variance across targets was much smaller, hence the significant paired *t*-test results).

#### Attractiveness ratings of photos by independent raters

Attractiveness ratings for the six raters (three females) showed adequate interrater reliability. T1: female raters (α = 0.71), male raters (α = 0.84); T2: female raters (α = 0.62), male raters (α = 0.82). There were no significant differences in facial attractiveness at T1 (partner [*M* = 4.84, SD = 1.34] vs. stranger images [*M* = 4.86, SD = 0.76], *t*_42_ = 0.11, *P* > 0.10) nor at T2 (partner [*M* = 5.93, SD = 1.18] vs. stranger images [*M* = 5.98, SD = 0.96], *t*_41_ = 0.36, *P* > 0.10).

### Covariation of SPS with neural activity in response to partners' and strangers' emotions

First, we compared neural responses to *emotional* (happy, sad) versus *neutral* expressions for each target (e.g., partner happy vs. neutral; stranger happy vs. neutral). Because the neutral condition was only included at T2, these analyses are restricted to T2 data. Tables 1 and 2 show results for positive emotions and negative emotions, respectively.

**Table 1:** Associations of sensory processing sensitivity with regional brain activity in response to partners' happy (vs. neutral) and strangers' happy (vs. neutral) facial images

Brain region	Left				Right			
		
Region-of-interest results	*x*	*y*	*z*	*P*, cluster	*x*	*y*	*z*	*P*, cluster
Partner happy versus partner neutral: positive association								
Anterior insula					36	21	6	0.006, 54
Inferior frontal gyrus	−42	24	3	0.025, 8	48	27	6	0.001, 86
Angular gyrus					63	−51	12	0.023, 17
Superior parietal lobe					36	−39	42	0.002, 29
Temporoparietal junction					51	−54	21	0.006, 38
Middle temporal gyrus	−48	−54	9	0.019, 30				
Middle/superior temporal cortex					60	−61	21	0.010, 39
Superior temporal sulcus	−54	−54	3	0.038, 65	48	−54	9	0.019, 26
Dorsolateral prefrontal cortex					42	39	21	0.008, 42
Cingulate cortex					3	24	42	0.009, 58
Cingulate					6	6	57	0.006, 43
Premotor area					48	6	54	0.002, 23
Presupplementary motor area					6	18	54	0.003, 36
Superior occipital gyrus/precuneus					30	−72	39	0.007, 45
Precuneus	−15	−75	21	0.022, 15				
Middle occipital gyrus	−51	−69	−6	0.005, 55	39	−75	−12	0.005, 41
Inferior occipital cortex	−57	−66	−3	0.011, 44				
Stranger happy versus stranger neutral: positive association								
Anterior insula/inferior frontal gyrus					27	27	0	0.004, 6
Anterior insula					27	27	−6	0.004, 19
Inferior frontal gyrus					27	27	−2	0.004, 7
Middle temporal gyrus					51	6	−24	0.013, 9
Premotor cortex	−33	27	12	0.016, 8				
Precentral gyrus	−63	−3	18	0.005, 27	45	−12	24	0.002, 11
Hippocampus	−27	−9	−15	0.002, 25				
Amygdala/anterior hippocampus	−27	−9	−12	0.002, 5				
Whole-brain results								
Medial prefrontal cortex	−18	30	−12	<0.001, 35				
Subcallosal cingulate	−9	54	−12	<0.001, 60				

Results are for brain activations associated with greater Highly Sensitive Person scale scores (controlling for neuroticism scores). MNI coordinates (*x*, *y*, *z*) are at the maximum value for the cluster, which may be elongated in any direction. For ROIs, *P* values are for small volume correction with *P*(unc) <0.05. Cluster = cluster size. For whole-brain results, we applied *P* < 0.001 (uncorrected for multiple comparisons) with a spatial extent of >15 contiguous voxels. AG, angular gyrus; AI, anterior insula; DLPFC, dorsolateral prefrontal cortex; IFG, inferior frontal gyrus; mPFC, medial prefrontal cortex; MTG, middle temporal gyrus; PMA, premotor area; pSMA, presupplementary motor area; TPJ, temporoparietal junction.

**Table 2:** Associations of sensory processing sensitivity with regional brain activity in response to partners' sad (vs. neutral) and strangers' sad (vs. neutral) facial images

Brain region	Left				Right			
		
Region-of-interest results	*x*	*y*	*z*	*P*, cluster	*x*	*y*	*z*	*P*, cluster
Partner sad versus partner neutral: positive association								
Anterior insula					33	18	−3	0.038, 10
Anterior intraparietal sulcus					36	−39	45	0.029, 8
Inferior parietal cortex					45	−27	54	0.012, 19
Middle temporal gyrus	−42	−66	9	0.019, 20	36	−63	−3	0.010, 29
Superior temporal sulcus	−51	−45	15	0.037, 3	51	−45	12	0.037, 7
Dorsolateral prefrontal cortex					42	39	21	0.010, 32
Cingulate					6	6	57	0.029, 11
Caudate					9	−3	30	0.011, 19
Premotor cortex					45	3	33	0.008, 45
Premotor area					27	3	54	0.014, 14
Postcentral gyrus					48	−27	57	0.011, 49
Claustrum					36	15	−6	0.016, 10
Stranger sad versus stranger neutral: positive association								
Middle temporal gyrus					48	−48	−6	0.005, 17
Middle temporal gyrus					12	−9	−15	0.016, 13
Supramarginal gyrus					39	−42	30	0.008, 17
Hippocampus/parahippocampus					33	−15	−21	0.017, 12
Premotor area	−33	27	15	0.029, 14				
Cingulate gyrus	−18	6	30	0.009, 20				
Thalamus	−3	−33	3	0.009, 7				
Stranger sad versus stranger neutral: negative association								
Occipital					3	−68	9	<0.001, 52
Middle temporal gyrus	−48	−48	0	<0.001, 86				

Results are for brain activations associated with greater Highly Sensitive Person scale scores (controlling for Neuroticism scores). MNI coordinates (*x*, *y*, *z*) are at the maximum value for the cluster, which may be elongated in any direction. For ROIs, *P* values are for small volume correction with *P*(unc) <0.05. Cluster = cluster size. AI, anterior insula; DLPFC, Dorsolateral prefrontal cortex; MTG, middle temporal gyrus; PMA, premotor area.

#### Partner happy versus partner neutral

For the partner happy versus neutral contrast, ROI analyses showed significant positive associations for greater HSP scores with brain activations in a number of areas as shown in Table 1. Bilateral findings were seen in the inferior frontal gyrus (IFG), superior temporal sulcus, and middle occipital gyrus. Right-hemisphere findings were in the anterior insula (AI), angular gyrus (AG), superior parietal lobe (SPL), temporoparietal junction (TPJ), middle/superior temporal cortex, dorsolateral prefrontal cortex (DLPFC), cingulate cortex/cingulate, premotor area (PMA), presupplementary motor area (pSMA), and superior occipital gyrus/precuneus. Left-hemisphere findings were in the middle temporal gyrus (MTG), precuneus, and inferior occipital cortex. There were no significant negative associations or any exploratory findings for this contrast.

#### Stranger happy versus stranger neutral

ROI analysis showed significant positive associations for HSP scores with brain activations in response to stranger happy versus stranger neutral images as shown in Table 1. Bilateral activations were found in the precentral gyrus; right-hemisphere activations in the AI, IFG, and MTG; and left-hemisphere activations in the premotor cortex, hippocampus, parahippocampal gyrus, and in the area of the anterior hippocampus/amygdala. Exploratory, whole-brain analyses showed positive associations in the left-medial prefrontal cortex (mPFC) and subcallosal cingulate. There were no significant negative associations.

#### Overlapping activations for partner and stranger happy stimuli

Both the partner happy (vs. neutral) and stranger happy (vs. neutral) conditions showed activations of the right AI and IFG in similar regions. Activations of the PMA and MTG also appeared in both contrasts, but in opposite hemispheres and in slightly different areas.

#### Partner sad versus partner neutral

ROI analysis showed significant positive associations for greater HSP scores with brain activations in response to partner sad versus neutral images as shown in Table 2. Bilateral activations were seen in the MTG and superior temporal sulcus; right-hemisphere activations in the AI, anterior intraparietal sulcus, inferior parietal cortex, DLPFC, cingulate, caudate, premotor cortex, PMA, postcentral gyrus, and claustrum; and no significant left-hemisphere localized activations. There were no significant negative associations or whole brain, exploratory findings.

#### Stranger sad versus stranger neutral

ROI analysis showed significant positive associations for HSP scores with brain activations in response to stranger sad versus neutral images as shown in Table 2. Right-hemisphere activations were found in the MTG, supramarginal gyrus, and the hippocampus/para-hippocampus; in the left-hemisphere PMA, cingulate gyrus, and the thalamus. Whole brain, exploratory analyses showed negative associations (greater SPS scores associated with less neural activation) in the right occipital lobe and in the left MTG.

#### Overlapping activations for partner and stranger sad facial expressions

Both the partner sad versus neutral and stranger sad versus neutral conditions showed activation of the right MTG. Activation of the PMA also appeared in both contrasts, but in opposite hemispheres and in slightly different areas.

#### Covariation of SPS with neural activity to partners' versus strangers' emotions

In the next series of analyses, we examined the link between HSP scores and neural responses to emotions expressed by partners versus strangers (e.g., partner happy vs. stranger happy). These analyses were conducted at both T1 and T2, providing a replication.

#### Partner happy versus stranger happy at T1

**Figure 3:**
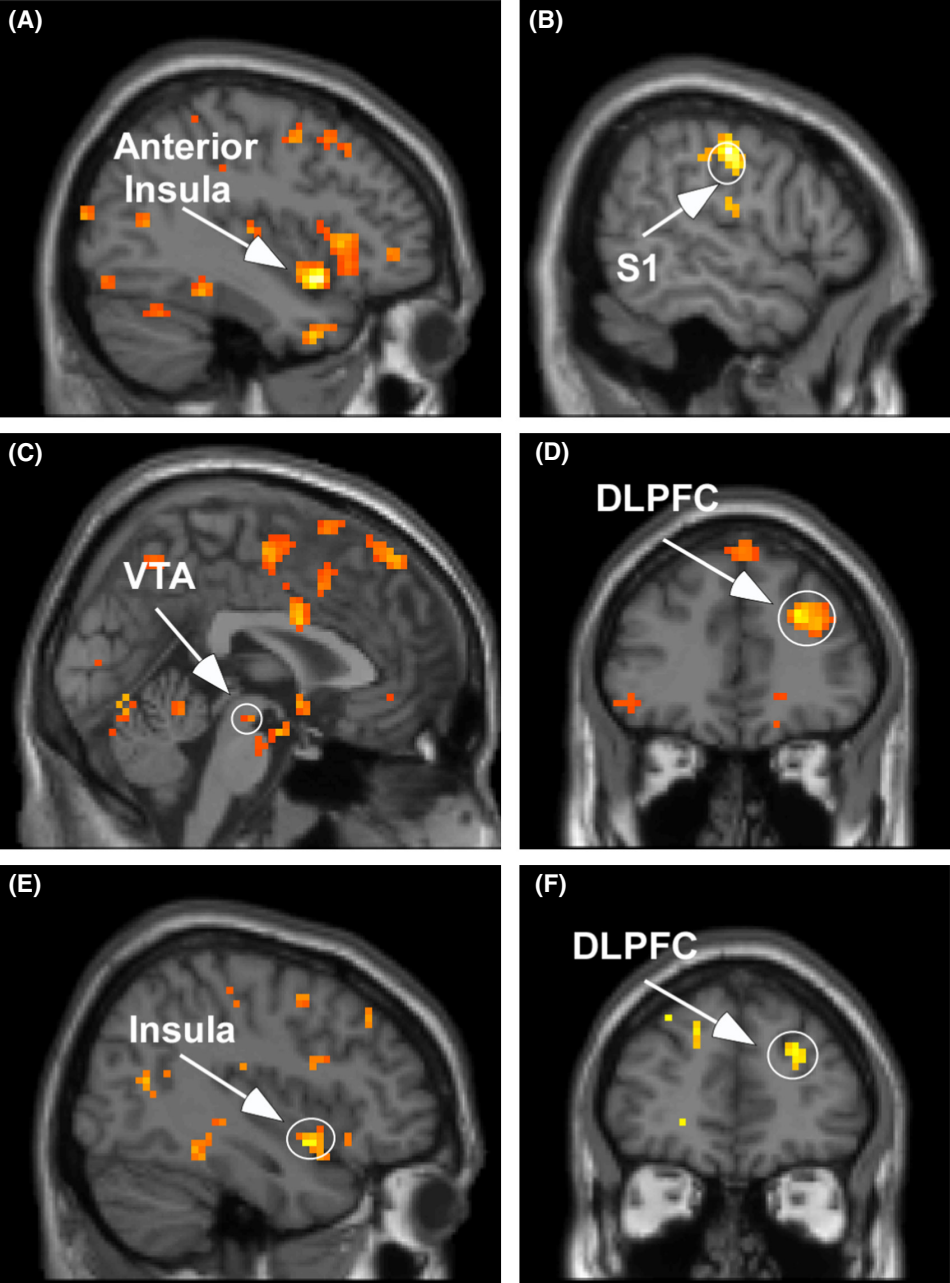
Images showing brain activations significantly associated with higher scores on the Highly Sensitive Person (HSP) scale scores (controlling for neuroticism scores) at Time 1 for the partner happy versus stranger happy condition in the (A) anterior insula (AI), (B) primary somatosensory cortex (S1), (C) ventral tegmental area (VTA), and (D) dorsolateral prefrontal cortex (DLPFC); and for the partner sad versus stranger sad condition in the (E) insula and (F) the DLPFC.

ROI analysis showed significant positive associations for HSP scores with brain activations for the partner happy versus stranger happy condition as shown in Table 3, section 1. Bilateral activations resulted in the insula, anterior parietal region, and the PMA/supplementary area; right-hemisphere activations in the AI, IFG, AG, SPL, BA 5,7/intraparietal sulcus, parietal operculum, DLPFC, premotor cortex, superior frontal gyrus, and the primary somatosensory cortex, and the ventral tegmental area (VTA) (MNI: 2, −19, −15, cluster = 3, as shown in Fig. 3C); and left-hemisphere activations in the mPFC and MTG. There were no significant negative associations or whole-brain findings for this contrast.

**Table 3:** Associations of sensory processing sensitivity with regional brain activity in response to partner versus stranger facial images across time points

	Left				Right			
		
Brain region	*x*	*y*	*z*	*P*, cluster	*x*	*y*	*z*	*P*, cluster
Partner happy versus stranger happy: positive associations								
Insula	−33	12	6	0.002, 19	36	21	−12	0.003, 41
Anterior insula/inferior frontal gyrus^*^^1997^					45	27	21	0.006^*^^1997^, 29
Angular gyrus^*^^1997^					34	−72	28	<0.001^*^^1997^, 22
Anterior parietal region^*^^1997^	−27	−48	66	0.015, 13	27	−48	72	0.010^*^^1997^, 28
Superior parietal lobe^*^^1997^					16	−63	63	0.005^*^^1997^, 38
Superior parietal lobe/intraparietal sulcus					33	−45	54	0.031, 21
Parietal operculum					52	−22	30	0.001, 47
Dorsolateral prefrontal cortex^*^^1997^					36	39	27	0.007^*^^1997^, 17
Medial prefrontal cortex	−9	66	17	0.012, 6				
Premotor cortex^*^^1997^					54	9	48	0.022^*^^1997^, 18
Premotor/supplementary area^*^^1997^	−9	−3	51	0.001, 29	24	3	57	<0.001^*^^1997^, 48
Superior frontal gyrus^*^^1997^					9	9	60	0.006^*^^1997^, 14
Primary somatosensory cortex					48	−18	48	<0.001, 19
Primary somatosensory cortex					57	−15	42	<0.001, 29
Middle temporal gyrus^*^^1997^	−45	−69	9	0.027^*^^1997^, 5				
Partner sad versus stranger sad: positive associations								
Insula	−33	18	9	0.019, 29	42	24	−12	0.025, 28
Insula^*^^1997^	−42	−33	21	0.007^*^^1997^, 9				
Anterior parietal region	−27	−48	66	0.006, 7				
Superior parietal lobe					12	−57	60	0.009, 29
Superior parietal lobe/intraparietal sulcus					27	−45	51	0.002, 5
Superior frontal gyrus^*^^1997^	−9	18	48	0.011^*^^1997^, 15				
Dorsolateral prefrontal cortex					27	45	27	0.002, 45
Premotor area^*^^1997^	−6	−3	57	0.001, 34	27	3	54	0.009^*^^1997^, 16
Cingulate^*^^1997^					12	6	60	0.001^*^^1997^, 37
Cingulate gyrus^*^^1997^	−4	11	29	0.002, 19	10	3	45	0.002^*^^1997^, 52
Thalamus	−3	−21	0	<0.001, 15				
Partner sad versus stranger sad: negative association at T1								
Lateral orbitofrontal cortex					45	45	−6	<0.001, 62
Inferior frontal gyrus					30	35	12	<0.001, 40

Results are for brain activations associated with greater Highly Sensitive Person scale scores (controlling for Neuroticism scores). MNI coordinates (*x*, *y*, *z*) are at the maximum value for the cluster, which may be elongated in any direction. For ROIs, *P* values are for small volume correction with *P*(unc) <0.05. Replications at T2 are indicated by a “^*^”.AG, angular gyrus; AI, anterior insula; DLPFC, Dorsolateral prefrontal cortex; IFG, inferior frontal gyrus; mPFC, medial prefrontal cortex; MTG, middle temporal gyrus; PMA, premotor area.

#### Partner happy versus stranger happy at T2

ROI analyses at T2 replicated activations for the 13 of the 18 individuals scanned at T1 for the partner happy versus stranger happy contrast in the right hemisphere of the AI/IFG, AG, anterior and superior parietal regions, DLPFC, premotor cortex, PMA, and the cingulate; and in the left hemisphere MTG. Findings replicated at T2 are indicated by a “*” in Table 3, section 1.

#### Partner sad versus stranger sad at T1

ROI analysis showed significant positive associations for HSP scores with brain activations for the partner sad versus stranger sad contrast as shown in Table 3, section 2. Bilateral activations were found in the insula, PMA, and cingulate gyrus; right-hemisphere activations in the SPL, intraparietal sulcus, DLPFC, and the cingulate; and left-hemisphere activations in the anterior parietal region, superior frontal gyrus, and the thalamus. As shown in Table 3, section 3, whole brain, exploratory analyses revealed negative associations of greater HSP scores with less neural activation in the right hemisphere of the lateral orbitofrontal cortex and the IFG.

#### Partner sad versus stranger sad at T2

ROI analysis showed several replications at T2 for the 13 of the 18 individuals scanned at T1 for the partner sad versus stranger sad contrast as indicated by a “*” in Table 3, section 2. Overlapping activations were found in the left hemisphere insula and superior frontal gyrus; right hemisphere PMA, cingulate, and the cingulate gyrus. Negative associations did not replicate at T2. However, exploratory whole-brain analyses revealed significant activation of the subcallosal area (MNI coordinates: 12, 39, −3, cluster = 9) in association with lower HSP scores.

#### Common findings across all contrasts

Across all conditions, we found activation of the PMA and premotor cingulate in association with HSP scores (controlling for neuroticism).

## Discussion

Sensory processing sensitivity is proposed to be an innate trait associated with greater sensitivity to environmental and social stimuli (e.g., Aron et al. 2012). Behaviorally it is characterized by more elaborate processing of stimuli and, to facilitate this, “pausing to check” before approaching novel situations. Theory and research suggest that emotional relevance guides this more extensive processing such that socially relevant stimuli tend to evoke stronger reactions in those higher on the trait. Thus, we examined individuals' neural activity in response to perceiving others' emotional expressions as a function of SPS. Across two time points, we scanned the brains of newly married individuals while they viewed photos of their partners and strangers displaying either happy or sad, and at T2, also neutral, facial expressions. This enabled us to investigate whether individuals with greater SPS (assessed by the HSP scale, the standard measure of SPS) would show stronger activations in brain regions reflecting awareness, empathy, and motor control in response to others' emotions. Inclusion of different stimuli also allowed us to examine whether individuals higher on SPS would show stronger brain activations to the emotional displays (a) of close others (vs. strangers) and (b) for positive (vs. negative or neutral) emotions, as suggested by theory and some self-report studies.

As predicted, greater HSP scores were associated with stronger activations of brain regions involved in awareness, integration of sensory information, empathy, and preparation for action in response to emotionally evocative social stimuli. Our results also supported additional predictions: we found stronger activations in response to close others and to positive social stimuli, including activation in the VTA, a dopamine-rich area well-known for its involvement in reward processing. These findings are consistent with research showing that emotional bonds between actors and perceivers facilitate mutual intention perception, and those with stronger bonds show greater intention understanding (e.g., Ortigue and Bianchi-Demicheli 2008). Our results were also in-line with previous SPS studies showing strong responses to positive stimuli (Jagiellowicz 2012). It is interesting to note that in our study we found no evidence of activation in the amygdala—a brain region that is known to be involved in emotional processing—as a function of SPS in response to emotional social stimuli. These results suggest that at least when viewing emotionally evocative photographs, SPS does not necessarily engage limbic emotional processes but rather influences preparations to act via higher order systems involved in awareness, integration of sensory information, and action planning.

### The highly sensitive brain: alert and ready to respond

Across all possible conditions we found positive associations with HSP scores (controlling for neuroticism) in the cingulate and PMA, regions involved in attention and action planning. These findings are robust, as we varied the target (partner vs. stranger) and emotional display (positive, negative, and neutral) of our stimuli, and replicated findings after 1 year for the subset of the original sample that was rescanned. Also, results in the PMA replicated findings from a previous fMRI study of SPS measuring responses to landscape images (Jagiellowicz et al. 2011).

The cingulate area found in this study was very similar to that reported in a meta-analysis of 40 empathy studies (Fan et al. 2011). The cingulate is important for the recognition of others' actions, in both humans and other primates (e.g., Rizzolatti et al. 1996), and in conjunction with the insula (another area activated in association with SPS, see below) it appears to be involved in moment-to-moment awareness (Craig 2009). A review of the studies on cingulate function suggests that it is an area where motor control, cognition, and drive (or arousal) interface (Paus 2001). In the present context, activation of the cingulate may reflect greater attention and alertness in response to socially relevant stimuli consistent with SPS theory.

The PMA, also found across all conditions in this study, is involved in unconscious behavioral control and action planning (e.g., Cross et al. 2006). It is responsible for action preparation, guidance, and direct control of movements (Graziano 2006), and through connections with the PFC, it is key site for behavioral control. Activation of the PMA in this study is consistent with SPS theory and research which propose that SPS is characterized by behaviors such as “pausing to check” (vs. approaching quickly).

Another notable activation found for many conditions (for partners and strangers and for both happy and sad facial expressions) appeared in the MTG—a region that is important for emotional meaning making (e.g., Murty et al. 2011) and described as a “semantic hub” for language, visual, and auditory processing (e.g., Dronkers et al. 2004; Binder et al. 2009). Stronger activation of the MTG in association with greater HSP scores is consistent with SPS theory and research showing that individuals higher on the trait display greater awareness and responsivity to a variety of stimuli, including loud noises, bright lights, strong smells, and others' moods.

Collectively, the present results support the notion that SPS is a trait associated with enhanced awareness and responsiveness to others' moods as it engages brain systems involved in sensory information processing and integration, action planning, and overall awareness. These findings highlight how the highly sensitive brain mediates greater attunement and action planning needed to respond to the environment, particularly relevant social contexts.

### The highly sensitive brain: empathy and integration of others' emotions

Across most of our conditions (except stranger sad versus neutral contrast) we found that HSP scores were positively associated with activation of the insula, implicated in limbic functions, sensorimotor integration, and a wide range of functions including attention, emotion, and self-referential processing (e.g., Phan et al. 2002; Jabbi and Keysers 2008; Cauda et al. 2011). Activations in this study were found in an area similar to that reported in two meta-analyses of 40 and 32 empathy studies and one study involving perception of a romantic partner's pain (Singer et al. 2004; Fan et al. 2011; Lamm et al. 2011).

The insula shows connectivity with other regions of the brain associated with emotion detection and interpretation, such as the IFG (which was found for all HSP associations for positive emotion conditions in this study). The IFG is proposed to be part of a Mirror Neuron System (MNS) (e.g., Iacoboni et al. 1999; Jabbi and Keysers 2008; Van Overwalle and Baetens 2009) that permits humans to rapidly and intuitively sense others' goals and intentions (e.g., Cross et al. 2006; Van Overwalle and Baetens 2009). Primates' IFG neurons fire both when they perform and observe hand actions (e.g., Rizzolatti and Craighero 2004; Nelissen et al. 2005). Numerous studies have shown activation of the IFG in the same area for the observation and execution of movements (e.g., Decety et al. 1997), suggesting its importance in imitation-learning and understanding others' intentions (Gallese and Goldman 1998). These results suggest that highly sensitive individuals “feel” and integrate sensory information to a greater extent in response to their close others' affective states, in particular positive emotional states (relative to a nonclose other's and to neutral affect).

Somewhat similarly, high sensitivity was associated with stronger activation of the AG in response to five of the six partner conditions (the exception was the T2 partner sad versus neutral contrast). AG has been implicated in self-representation, understanding of metaphors, cognition (specifically internal dialog), and abstract representation of the self (e.g., Blanke et al. 2002; Arzy et al. 2006). Activation of the AG has also been shown in several fMRI studies of romantic love (e.g., Ortigue et al. 2007; Acevedo et al. 2011). Taken together (along with activation of the IFG), this study suggests how the brain, via brain circuits important for integration of others' states and empathy, mediates the experiences of highly sensitive individuals as being more responsive to others' moods.

Furthermore, highly sensitive individuals showed stronger activation in the VTA for the partner happy versus stranger happy contrast, but not for the other contrasts. The VTA has been shown to be activated in response to several positive stimuli and in other studies of romantic partners (e.g., Aron et al. 2005b; Acevedo et al. 2011). The finding that it is more active under an emotional condition herein is consistent with the idea that sensitive people are more responsive to emotional and positive stimuli.

Finally, highly sensitive individuals showed stronger activation of the DLPFC across most partner contrasts. The DLPFC is involved in higher order cognitive processing, decision making, and complex tasks. We speculate that significant activation of the DLPFC, specifically in response to socially relevant stimuli, reflects the greater depth and higher order processing (Miller 2000) consistent with behavioral descriptions of high-SPS individual's greater conscientiousness and responsiveness to others' moods (Aron et al. 2012).

### Is SPS selective?

SPS may be evolutionary advantageous under some conditions, but it is still metabolically costly, so selective attention to close others may be a way to conserve energy. Although SPS is expected to increase response to environmental stimuli in general (especially socially relevant emotional stimuli), the inclusion of both happy and sad faces permitted us to test emotional responses more broadly, and examine the possibility that SPS might be especially strongly associated with positive emotions, given previous findings noted in the Introduction.

This study suggests that highly sensitive individuals show similar patterns of neural activation for partner happy and sad (vs. neutral) facial expressions, and also for happy strangers (vs. neutral) in areas implicated in empathy, sensorimotor integration (e.g., the insula and IFG). However, these activations did not appear in the stranger sad (vs. neutral) condition. Hence, SPS seems to be a selective trait, whereby partners' emotional expressions are given priority. In addition, stronger brain activation of the insula and IFG in response to all happy conditions, including the happy strangers are worth noting, perhaps supporting the particular susceptibility to positive environments.

When partner and stranger were directly contrasted, highly sensitive individuals showed stronger brain activations in brain regions known to be involved in self-other processing (e.g., the AG) in response to partners' facial expressions (including stronger reactions to partners' happy expressions) than to strangers. When directly comparing activations to partner happy versus stranger happy faces, highly sensitive individuals also showed stronger activation of regions involved in empathy, self-other processing, decision-making, integration of sensory information, and action planning (e.g., in the insula, IFG, AG, SPL, DLPFC, PMA, cingulate, and MTG).

Although the above argues for a difference between partner and stranger, there is at the same time the interesting result that activation in areas related to imitation and self-other processing (IFG and AG) was somewhat similar for the partner happy versus neutral condition and stranger happy versus neutral (but not for stranger sad vs. neutral), suggesting a bias toward positive expressions. Greater SPS was also associated with stronger activation of brain regions involved in attention, empathy, higher order cognitive processing, and action planning in response to close others (vs. strangers), and particularly to their positive emotions (vs. negative and neutral).

Collectively, these findings suggest that SPS may be a selective strategy and that for some evolutionary reason, such as conservation of metabolic resources, highly sensitive individuals process information about close others and positive emotions more thoroughly. Perhaps this greater response to close others' positive emotions explains their unusual susceptibility to positive social environments (Pluess and Belsky 2013). Whether learned or innate, individuals with greater SPS appear to be reducing their reactions to negative emotional information that may not be particularly salient, as for strangers versus close others. This is consistent with behavioral evidence of highly sensitive individuals reporting that they tend to avoid negative overstimulation (such as loud sirens, horror movies, and having too much to do at once) and needing recovery time after viewing arousing stimuli. Nevertheless, activation of regions involved in awareness, higher order processing, and action planning suggest that HSPs are attentive and preparing to respond to their partner's needs when happy or sad.

## Future Directions and Limitations

Our sample consisted of individuals soon-to-be or recently married. Thus, responses to partners' may reflect particularly strong activations, as the early stages of marriage tend to be emotionally charged, and particularly positive around the time of the wedding. This may not be indicative of responses to others in general, close others, and across relationship stages. However, we also examined responses to strangers' images and partner's neutral images, and data with the same group of individuals 1 year after the first scan, thus providing strong evidence for the pattern of activations. Nevertheless, future research may aim to examine SPS across other important relationships (e.g., parent–child), relationship stage, and individuals with more diverse socioeconomic status and ethnicity.

Although the present results support existing SPS theory and research, there are limitations. First, as mentioned previously, our sample was largely homogenous, constraining the generalizability of our results to other populations. Second, the only measure of SPS was the HSP scale. As more nonself-report measures become available, it would be important to use these in such studies. Third, as our postscan emotion ratings were collected after the scanning session (although participants were still in the scanner), it is possible that emotional states elicited during the experiment were subject to recall effects or dampened at the time of inquiry. Fourth, fMRI research, in general, implies several inferences such that the labeling of some brain regions as “empathy” areas oversimplifies the complex neural circuitry probably involved. However, we attempted to highlight the variety of functions across some key sites. In addition, as we had numerous ROIs, for each region we applied small volume corrections independently. This increases the likelihood of finding a positive result; therefore, we applied FDR to each ROI (which corrects for multiple comparisons), but report the uncorrected *P* values to acknowledge this limitation. Finally, fMRI studies in general do not demonstrate that any such region is the cause of an experience (vs. that the experience is the cause of the activation). Thus, it is best to be conservative in interpretation of results provided herein as telling us how the brain creates responses.

## Conclusion

The primary goal of this study was to extend research on SPS by examining the brain activations engaged in processing emotional social stimuli. Using fMRI we measured the brain activity of participants in response to positive and negative facial images of their partners and strangers in two studies, providing a replication. Across all conditions, results showed activation of brain regions involved in awareness, attention, and action planning (in the cingulate and PMA), replicating results from a previous fMRI study of SPS measuring responses to landscape images. Other robust neural activations (appearing in most conditions) were found in regions implicated in the integration of sensory information, emotional meaning making, and empathy. Additional notable results for SPS were found in regions implicated in self-other processing, the mirror neuron system, self-awareness, and higher order cognitive processing. These responses were shown for both partners and strangers, but also showed some selectivity for partners and for positive emotions. The present findings support SPS theory and research suggesting that it is a trait associated with enhanced awareness and behavioral readiness to respond to salient environmental stimuli, particularly important social situations. These results highlight how the highly sensitive brain may mediate greater attunement to others' and responsiveness to others' needs.

## Supporting Information

**Table S1.** Regions of interest (ROIs) used to examine regional brain activations for an fMRI study of sensory processing sensitivity.
