# Supplementary material for: The highly sensitive brain: an fMRI study of sensory processing sensitivity and response to others' emotions
Source: Brain Behav. 2014 Jun 23;4(4):580–94. doi: 10.1002/brb3.242 (PMC4086365; doi:10.1002/brb3.242)
Supplement: Supplementary file 1 — Table S1. Regions of interest (ROIs) used to examine regional brain activations for an fMRI study of sensory processing sensitivity. [file brb30004-0580-SD1.doc]

**Supplementary Table 1**

*Regions of Interests (ROIs) Used to Examine* Regional Brain Activations for an fMRI Study of Sensory Processing Sensitivity

|  |  | Talairach Coordinates |  | Seed Paper |
| --- | --- | --- | --- | --- |
| Region | x | y | z |  |
| Amygdala/anterior hippocampus | -27 | -9 | -12 | 7 |
| Angular gyrus | +34 | -72 | 28 | 1 |
| Angular gyrus | +63 | -51 | 12 | 13 |
| Anterior insula | +27 | 27 | -6 | 4 |
| Anterior insula | +36 | 21 | 6 | 5 |
| Anterior intraparietal sulcus | +36 | -39 | 45 | 10 |
| Anterior insula/IFG | +45 | 27 | 21 | 8 |
| Anterior parietal region | -27 | -48 | 66 | 9 |
| Anterior parietal region | +27 | -48 | 72 | 9 |
| Caudate | +9 | -3 | 30 | 7 |
| Cingulate | +6 | 6 | 57 | 4 |
| Cingulate cortex | +3 | 24 | 42 | 4 |
| Cingulate gyrus | -4 | 11 | 29 | 2 |
| Cingulate gyrus | +10 | 3 | 45 | 2 |
| Claustrum | +36 | 15 | -6 | 7 |
| DLPFC | +42 | 39 | 21 | 5 |
| Dorsolateral prefrontal cortex | +42 | 39 | 21 | 5 |
| Hippocampus | -27 | -9 | -15 | 5 |
| Hippocampus/ parahippocampus | +33 | -15 | -21 | 7 |
| IFG | +50 | 21 | 23 | 9,12 |
| Inferior frontal gyrus | -42 | 24 | 3 | 4,9,11 |
| Inferior frontal gyrus | +48 | 27 | 6 | 4,9,11 |
| Inferior occipital cortex | -57 | -66 | -3 | 5 |
| Inferior parietal cortex | +45 | -27 | 54 | 5 |
| Insula | -42 | -33 | 21 | 3 |
| Insula | -39 | 18 | 3 | 4 |
| Insula | +36 | 24 | -12 | 4 |
| Insula | +39 | 9 | -12 | 9 |
| Middle occipital gyrus | -51 | -69 | -6 | 3,7 |
| Middle occipital gyrus | +39 | -75 | -12 | 3,7 |
| Middle temporal gyrus | +51 | 6 | -24 | 2 |
| Middle temporal gyrus | +12 | -9 | -15 | 7 |
| Middle temporal gyrus | -45 | -69 | 9 | 12 |
| Middle temporal gyrus | -42 | -66 | 9 | 7,12 |
| Middle temporal gyrus | +36 | -63 | -3 | 7,12 |
| Middle/superior temporal cortex | +60 | -61 | 21 | 5 |
| MPFC | -9 | 66 | 17 | 10 |
| Parahippocampal gyrus | -27 | -9 | -15 | 3 |
| Parietal operculum | +52 | -22 | 30 | 9 |
| Precentral gyrus | -63 | -3 | 18 | 2 |
| Precentral gyrus | +45 | -12 | 24 | 2 |
| Precuneus | -15 | -75 | 21 | 3 |
| Premotor area | +48 | 6 | 54 | 1,10 |
| Premotor area | -9 | -3 | 51 | 1,3 |
| Premotor area | +24 | 3 | 57 | 1,3 |
| Premotor area | -33 | 27 | 12 | 7 |
| Premotor cortex | +45 | 3 | 33 | 10 |
| Pre-supplementary motor area | +6 | 18 | 54 | 5 |
| Primary somatosensory cortex | +48 | -18 | 48 | 2 |
| Primary somatosensory cortex | +57 | -15 | 42 | 9 |
| Postcentral gyrus | +48 | -27 | 57 | 2 |
| SPL | +16 | -63 | 63 | 1 |
| SPL/ Intraparietal sulcus | +33 | -45 | 54 | 1 |
| Superior frontal gyrus | -9 | 18 | 48 | 4 |
| Superior occipital gyrus/ precuneus | +30 | -72 | 39 | 1 |
| Superior parietal lobe | +36 | -39 | 42 | 1,10 |
| Superior temporal sulcus | -54 | -54 | 3 | 10 |
| Superior temporal sulcus | +48 | -54 | 9 | 10 |
| Supramarginal gyrus | +39 | -42 | 30 | 3 |
| Temporoparietal junction | +51 | -54 | 21 | 10 |
| Thalamus | -3 | -33 | 3 | 3 |
| Ventral tegmental area | +2 | -19 | -15 | 1 |

**References**

^1^Aharon I, Etcoff N, Ariely D, Chabris CF, O’Connor E, and Breiter HC. Beautiful faces have variable reward value: fMRI and behavioral evidence. *Neuron* 32: 537–551, 2001. ^2^Bartels A and Zeki S. The neural basis of romantic love. *Neuroreport* 11: 3829–3834, 2000. ^3^Bartels A and Zeki S. The neural correlates of maternal and romantic love. *Neuroimage* 21: 1155–1166, 2004.
 ^4^Berns GS, McClure SM, Pagnoni G, and Montague PR. Predictability modulates human brain response to reward. *J Neurosci* 21: 2793–2798, 2001.

^5^Breiter HC, Gollub RL, Weisskoff RM, Kennedy DN, Makris N, Berke JD, Goodman JM, Kantor HL, Gastfriend DR, Riorden JP, Mathew RT, Rosen BR, and Hyman SE. Acute effects of cocaine on human brain activity and emotion. *Neuron* 19: 591–611, 1997.
 ^7^Delgado MR, Nystrom LE, Fissell C, Noll DC, and Fiez JA. Tracking the hemodynamic responses to reward and punishment in the striatum. *J Neurophysiol* 84: 3072–3077, 2000.
 ^8^Denton D, Shade R, Zamarippa F, Egan G, Blair-West J, McKinley M, Lancaster J, and Fox P. Neuroimaging of genesis and satiation of thirst and an interoceptor-driven theory of origins of primary consciousness. *Proc Natl Acad Sci USA* 96: 5304–5309, 1999. ^9^Elliott R, Friston KJ, and Dolan RJ. Dissociable neural responses in human reward systems. *J Neurosci* 20: 6159–6165, 2000. ^10^Elliott R, Newman JL, Longe OA, and Deakin JF. Differential response patterns in the striatum and orbitofrontal cortex to financial reward in humans: a parametric functional magnetic resonance imaging study. *J Neurosci* 23: 303–307, 2003.
 ^11^Elliott R, Newman JL, Longe OA, and William Deakin JF. Instrumental responding for rewards is associated with enhanced neuronal response in subcortical reward systems. *Neuroimage* 21: 984–990, 2004
 ^12^Garavan H, Pankiewicz J, Bloom A, Cho JK, Sperry L, Ross TJ, Salmeron BJ, Risinger R, Kelley D, and Stein EA. Cue-induced cocaine craving: neuroanatomical specificity for drug users and drug stimuli. *Am J Psychiatry* 157: 1789–1798, 2000.
 ^13^Knutson B, Adams CM, Fong GW, and Hommer D. Anticipation of increasing monetary reward selectively recruits nucleus accumbens. *J Neurosci* 21: RC159, 2001.
